# WNT/β-Catenin Signaling Promotes TGF-β-Mediated Activation of Human Cardiac Fibroblasts by Enhancing IL-11 Production

**DOI:** 10.3390/ijms221810072

**Published:** 2021-09-17

**Authors:** Edyta Działo, Marcin Czepiel, Karolina Tkacz, Maciej Siedlar, Gabriela Kania, Przemysław Błyszczuk

**Affiliations:** 1 Department of Clinical Immunology, Jagiellonian University Medical College, 30-663 Cracow, Poland; edyta.dzialo@doctoral.uj.edu.pl (E.D.); marcin.czepiel@uj.edu.pl (M.C.); karolina.tkacz@student.uj.edu.pl (K.T.); misiedla@cyf-kr.edu.pl (M.S.); 2 Center of Experimental Rheumatology, Department of Rheumatology, University Hospital Zurich, University of Zurich, 8952 Schlieren, Switzerland; gabriela.kania@uzh.ch

**Keywords:** cardiac fibroblasts, WNT3a, WNT5a, β-catenin, TGF-β signaling, IL-11, cardiac fibrosis

## Abstract

Cardiac fibrosis is a pathological process associated with the development of heart failure. TGF-β and WNT signaling have been implicated in pathogenesis of cardiac fibrosis, however, little is known about molecular cross-talk between these two pathways. The aim of this study was to examine the effect of exogenous canonical WNT3a and non-canonical WNT5a in TGF-β-activated human cardiac fibroblasts. We found that WNT3a and TGF-β induced a β-catenin-dependent response, whereas WNT5a prompted AP-1 activity. TGF-β triggered profibrotic signatures in cardiac fibroblasts, and co-stimulation with WNT3a or co-activation of the β-catenin pathway with the GSK3β inhibitor CHIR99021 enhanced collagen I and fibronectin production and development of active contractile stress fibers. In the absence of TGF-β, neither WNT3a nor CHIR99021 exerted profibrotic responses. On a molecular level, in TGF-β-activated fibroblasts, WNT3a enhanced phosphorylation of TAK1 and production and secretion of IL-11 but showed no effect on the Smad pathway. Neutralization of IL-11 activity with the blocking anti-IL-11 antibody effectively reduced the profibrotic response of cardiac fibroblasts activated with TGF-β and WNT3a. In contrast to canonical WNT3a, co-activation with non-canonical WNT5a suppressed TGF-β-induced production of collagen I. In conclusion, WNT/β-catenin signaling promotes TGF-β-mediated fibroblast-to-myofibroblast transition by enhancing IL-11 production. Thus, the uncovered mechanism broadens our knowledge on a molecular basis of cardiac fibrogenesis and defines novel therapeutic targets for fibrotic heart diseases.

## 1. Introduction

Cardiac fibrosis refers to the excessive accumulation of stromal cells and extracellular matrix (ECM) components in the myocardium and is often associated with cardiac pathologies. On the one hand, fibrotic scar develops following myocardial infarction to support tissue integrity and regeneration. On the other hand, hypertension, cardiomyopathy, local and systemic inflammation, co-morbidities, certain medications and aging may cause pathological tissue remodeling, affecting cardiac function [1]. Increased stiffness of cardiac tissue and disturbed propagation of electrical impulses are direct outcomes of fibrosis, and these changes may lead to heart failure and arrhythmias [2]. Consequently, patients with persistent cardiac fibrosis show longer hospitalization and increased mortality [3]. The prevalence of myocardial fibrosis varies depending on age and the occurrence of cardiovascular diseases. Late gadolinium-enhancement cardiac magnetic resonance identified fibrotic scars in up to one fourth of infarcted hearts [4], and in 31–38% of non-ischemic cardiomyopathy patients [5,6]. Furthermore, post-mortem histopathology studies reported fibrosis in every fifth heart of sudden cardiac death [7] and COVID-19 victims [8].

ECM-producing cardiac fibroblasts represent cell types responsible for the maintenance of the physiological architecture of the heart muscle. Once activated, cardiac fibroblasts play a central role in fibrotic processes in the heart. Activated cardiac fibroblasts show uncontrolled proliferation and ECM overproduction and may switch to the myofibroblast phenotype. Myofibroblasts combine high secretory potential with phagocytic and contractile properties [9,10]. Alpha-smooth muscle actin (αSMA)-positive stress fibers represent the most characteristic phenotypic feature of myofibroblasts in vitro. Cardiac injury or extracardiac triggers can activate the fibroblast-to-myofibroblast transition. On a molecular level, this activation is mediated by profibrotic signaling molecules and/or mechanical stress [1,9,11]

Transforming growth factor-beta (TGF-β) represents the most prominent profibrotic cytokine, which plays a key role in cardiac fibrosis. TGF-β triggers a phenotypic switch of cardiac fibroblast into myofibroblasts, and the disruption or blockade of TGF-β signaling was shown to protect from cardiac fibrosis in animal models [12]. Following binding of an active form of TGF-β to the transmembrane TGF-β type II receptor, the TGF-β type I receptor is recruited and activated. Canonical TGF-β receptor signal transduction involves Smad proteins and results in transcriptional response of Smad-responsive target genes [13]. In addition to Smad-dependent signaling, TGF-β activates pathways independently of Smads. TGF-β-activated kinase 1 (TAK1), phosphatidylinositol-3-kinase/Akt, RhoA–ROCK axis and mitogen-activated protein kinases p38, c-Jun N-terminal kinase (JNK) and extracellular signal-regulated kinase 1/2 (Erk1/2) represent examples of Smad-independent TGF-β signaling [14]. Despite the pivotal role of TGF-β in cardiac fibrosis, clinical use of direct or indirect TGF-β inhibitors is currently limited due to problems with toxicity [6,15].

TGF-β upregulates production of ECM components and multiple signaling molecules. Recent studies demonstrated that TGF-β controls production of profibrotic WNTs and interleukin 11 (IL-11) in cardiac fibroblasts [16,17]. In humans, the family of WNT ligands represents 19 proteins. Extracellular WNTs bind to transmembrane receptor complexes and activate β-catenin-dependent and β-catenin-independent downstream mechanisms [18,19]. In a canonical response, stimulation with certain WNTs, like WNT3a, leads to stabilization and nuclear translocation of β-catenin resulting in activation of the T-cell factor (TCF) and the lymphoid enhancer factor (LEF) transcription factors [20]. Other WNTs, like WNT5a, can trigger transcriptional responses independently of β-catenin. Such a non-canonical WNT response involves activation of Erk1/2 and JNK signaling pathways regulating the activity of the downstream activator protein 1 (AP-1) transcription factor, but also the RhoA–ROCK axis [21,22]. Thus, non-canonical WNTs and TGF-β control the same signaling pathways. Furthermore, TGF-β was shown to stimulate production and secretion of canonical WNTs and to activate β-catenin in cardiac fibroblasts [23,24]. Noteworthy, animal studies specifically pointed to the importance of the WNT/β-catenin pathway in cardiac fibrosis [25].

IL-11 represents a pleiotropic cytokine induced by respiratory viruses such as rhinovirus and parainfluenza, as well as diverse profibrotic factors including TGF-β, fibroblasts growth factor 2 (FGF2), endothelin 1 (EDN1) and platelet-derived growth factor (PDGF) [26,27]. Expression of IL-11 is strongly correlated with fibroblasts activation and associated with cardiac fibrosis.

Human cardiac fibroblasts-to-myofibroblasts transitions and development of cardiac fibrosis in mice were shown to be dependent on IL-11 signaling [17]. IL-11 plays an important profibrotic role not only in cardiac tissue but also in the lungs, kidney and liver [26,28]. A growing body of evidence points to IL-11 as a promising therapeutic target candidate in fibrotic disease. So far, however, little is known about molecular regulation of IL-11 production.

Herein, by using human cardiac fibroblasts, we studied the interplay between TGF-β and canonical WNT3a or non-canonical WNT5a in a fibrotic response. Our results indicate that WNT/β-catenin specifically controls profibrotic IL-11 in TGF-β-dependent fibroblast-to-myofibroblast transitions.

## 2. Results

### 2.1. Canonical and Non-Canonical WNT Responses in Human Cardiac Fibroblasts

**Figure 1:**
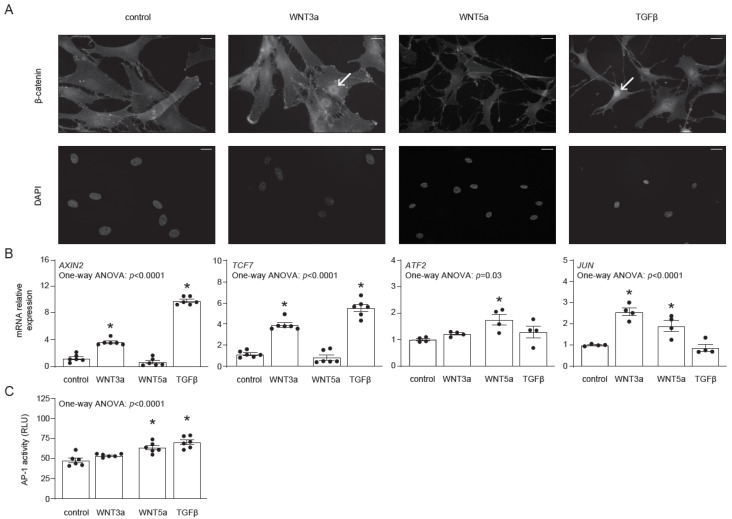
Activation of canonical and non-canonical WNT pathways in human cardiac fibroblasts. Human cardiac fibroblasts were stimulated with WNT3a, WNT5a or TGF-β. Panel (**A**) illustrates cellular localizations of β-catenin in fibroblasts 30 min after indicated treatment. DAPI stains cell nuclei. Arrows indicate nuclear localization of β-catenin. Data are representative of three independent experiments. Bar = 50 µm. Panel (**B**) represents expression of canonical (*AXIN2* and *TCF7*) and non-canonical (*JUN* and *ATF2*) WNT responsive genes 1 h after indicated treatment. Panel (**C**) shows quantification of AP-1 activity in AP-1-hCF reporter cardiac fibroblasts following 24 h treatment (*n* = 6). Graphs show means ± SEM, *p* values computed using one-way ANOVA followed by Dunnett’s multiple comparisons test, * *p* < 0.05 (post-hoc test vs. control).

Before investigating the effect of WNT signaling on the differentiation of human cardiac fibroblasts, we addressed their responsiveness to exogenous WNT3a and WNT5a. In this approach, we used the conditional medium of L-WNT3a, L-WNT5a (referred later as WNT3a and WNT5a, respectively) and of control L cell lines (used as controls for WNT3a and WNT5a), as well as TGF-β, which is known to induce endogenous WNT production. Nuclear translocation of β-catenin represents the most characteristic feature of the canonical WNT signaling. As expected, both WNT3a and TGF-β, but not WNT5a, induced nuclear translocation of β-catenin in cardiac fibroblasts (Figure 1A). In line with this finding, treatment with WNT3a or TGF-β induced expression of the β-catenin-responsive genes *AXIN2* and *TCF7* (Figure 1B), confirming activation of canonical WNT pathway. For the activation of the non-canonical WNT pathway, we analyzed the expression of *ATF2* and *JUN* and measured activity of the AP-1 transcription factor by using human cardiac fibroblasts transfected with the AP-1 reporter system (AP-1-hCF). WNT5a induced expression of *ATF2* and *JUN* in cardiac fibroblasts (Figure 1B). Furthermore, we observed significantly upregulated AP-1 activity in AP-1-hCFs following treatment with WNT5a or TGF-β (Figure 1C). Collectively, our data confirmed that human cardiac fibroblasts differentially respond to WNT3a and WNT5a, and that TGF-β could effectively activate canonical and non-canonical WNT pathways in these cells.

### 2.2. WNT3a and WNT5a Differentially Regulate Gene Expression in TGF-β-Activated Cardiac Fibroblasts

TGF-β plays a pivotal role in fibroblast activation. By employing full genome transcriptomics, we analyzed the transformation of human cardiac fibroblasts caused by TGF-β signaling. Differential gene expression analysis demonstrated that cardiac fibroblasts 72 h after treatment with TGF-β showed deregulated expression of 313 genes including genes associated with cell differentiation (GO:0030154, 124 genes, *p* ≤ 0.01), cellular component organization (GO:0051128, 92 genes, *p* ≤ 0.01) and extracellular matrix biology (GO:0031012, 41 genes, *p* ≤ 0.01). In particular, TGF-β induced expression of collagens (*COL1A1*, *COL5A1*, *COL16A1*, *COL11A1*) and other extracellular matrix proteins involved in fibrotic responses, including *POSTN*, *FN1*, *TNC* and *VCAN* (Supplementary Figure S1).

**Figure 2:**
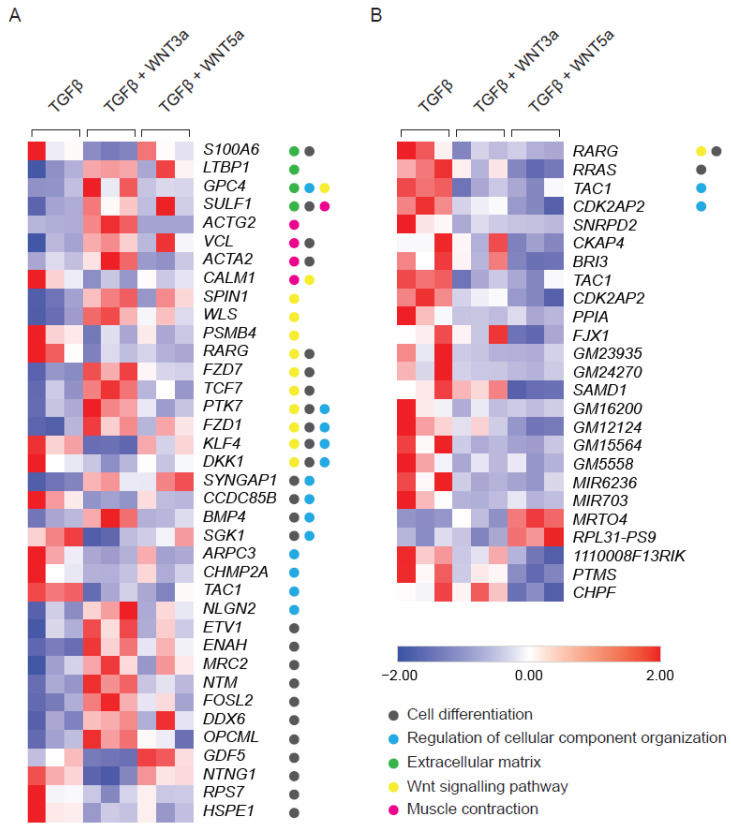
RNAseq analysis of TGF-β-activated fibroblasts following WNT3a or WNT5a treatment. Cardiac fibroblasts were stimulated with WNT3a or WNT5a in the presence of TGF-β for 72 h. Panel (**A**) shows a heat map of fibrosis-related genes differentially expressed (fold change > 2 and *p* < 0.05) in fibroblasts treated with WNT3a/TGF-β versus TGF-β alone (*n* = 3). All dysregulated genes of WNT3a/TGF-β versus TGF-β are presented in the Supplementary Figure S1. Panel (**B**) represents a heat map of all differentially expressed genes (fold change > 2 and *p* < 0.05) in cardiac fibroblasts treated with WNT5a/TGF-β versus TGF-β alone (*n* = 3). GO categories: Cell differentiation (GO:0030154), Regulation of cellular component organization (GO:0051128), Extracellular matrix (GO:0031012), WNT signaling pathway (GO:0016055) and Muscle Contraction (GO:0006936).

WNTs are typically produced by activated cells and act as secondary triggers. Therefore, the effect of exogenous WNTs was studied in TGF-β-activated fibroblasts additionally stimulated with WNT3a or WNT5a. Stimulation with WNT3a resulted in deregulation of 124 genes in TGF-β-treated fibroblasts (Supplementary Figure S2). Pathway analysis confirmed activation of WNT signaling (GO:0016055, 12 genes, *p* value ≤ 0.0001) and pointed to the induction of cell differentiation (GO:0030154, 26 genes, *p* value ≤ 0.0001) and cellular component organization (GO:0051128, 13 genes, *p* value ≤ 0.001). WNT3a specifically up-regulated genes involved in stress fibers formation, such as *ACTA2* (encoding alpha smooth muscle actin; αSMA), *ACTG2* (encoding gamma smooth muscle actin; αSMA) and *VCL* (encoding vinculin), as well as *LTBP1* (encoding latent-TGF-β-binding protein 1), which regulates TGF-β bioavailability (Figure 2A). In contrast to the profound effect of WNT3a, treatment with WNT5 upregulated expression of only 2 and downregulated 21 genes in TGF-β-activated cells (Figure 2B). Altogether, these results pointed to the relevance of the WNT canonical arm in enhancing TGF-β-mediated profibrotic responses in cardiac fibroblasts.

### 2.3. WNT/β-Catenin Signaling Promotes TGF-β-Dependent Fibroblast-to-Myofibroblast Transition

**Figure 3:**
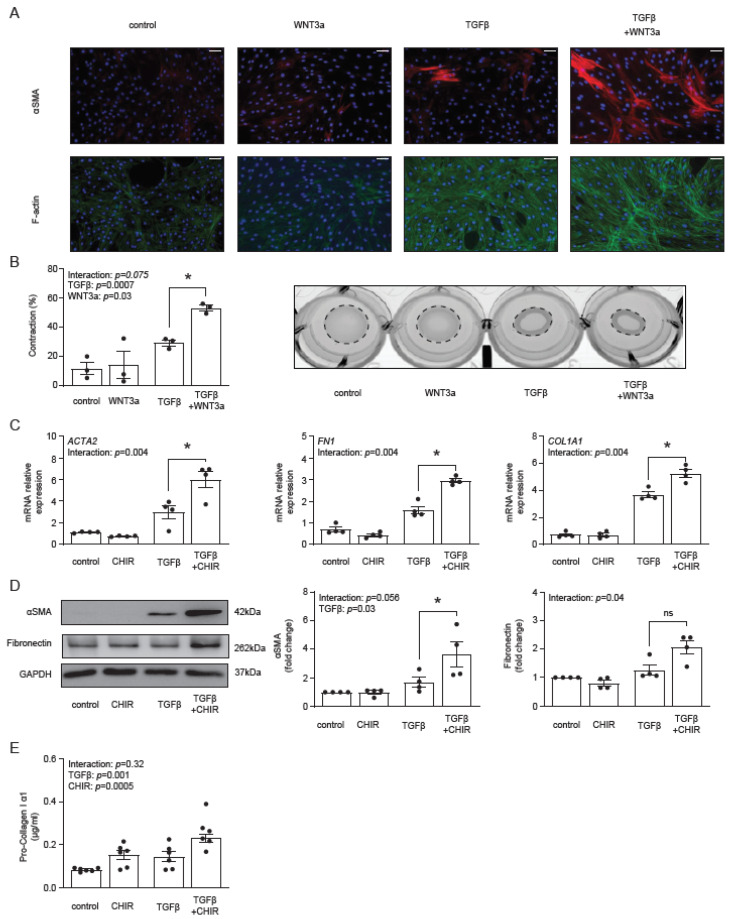
Panel (**A**) illustrates representative immunocytochemistry staining of αSMA, F-actin and cell nuclei (DAPI) of cardiac fibroblasts stimulated with WNT3a and/or TGF-β for 72 h. Data are representative of three biological independent experiments. Bar = 100 µm. Panel (**B**) shows quantification of gel contraction (72 h, *n* = 3) and representative image of collagen gel containing cardiac fibroblasts pre-treated as indicated. Panels (**C–E**) describe the profibrotic effect of GSK3β inhibitor CHIR99021 (CHIR) in the presence or absence of TGF-β in cardiac fibroblasts. Expression of profibrotic genes after 72 h of stimulation is presented in panel (**C**) (*n* = 4). Panel (**D**) shows representative immunoblots and the respective quantifications of αSMA and fibronectin after 72 h of stimulation (all *n* = 4) and panel (**E**) indicates pro-collagen I α1 level in supernatants (*n* = 6) stimulated for 5 days. Bars and whiskers represent means ±SEM. Main effect of WNT3a/CHIR, TGF-β and their interaction were tested using two-way ANOVA followed by Tukey’s multiple comparisons test, when the *p*-value for interaction was ≤ 0.1; * *p* < 0.05 (post-hoc TGF-β vs. TGF-β+WNT3a or CHIR vs. TGFβ+CHIR).

Our previous data showed that WNT3a stimulated production of αSMA and type I collagen in TGF-β-activated fibroblasts [29]. Following these results and the described above transcriptomics data, we measured the formation of αSMA-positive stress fibers by immunocytochemistry. WNT3a in the absence of TGF-β failed to increase the number of αSMA-positive cardiac fibroblasts, whereas in TGF-β-activated cells, WNT3a effectively boosted formation of αSMA-positive fibers (Figure 3A), which is a characteristic feature of myofibroblasts. Stress fibers are the major contractile structures in the cell; therefore, in the next step, we measured functional contraction of cardiac fibroblasts in a collagen gel. Obtained data confirmed that WNT3a enhanced contractile properties of TGF-β-activated fibroblasts (Figure 3B).

Next, we asked whether a specific pharmacological activation of the β-catenin-dependent pathway with the GSK3β inhibitor CHIR99021 could promote myofibroblast phenotype in TGF-β-activated cells. Treatment with CHIR99021 indeed up-regulated expression of *ACTA2*, *FN1* and *COL1A1* (Figure 3C), as well as increased the corresponding intracellular protein levels of αSMA, fibronectin (Figure 3D) and the amount of secreted pro-collagen I α1 (Figure 3E) in TGF-β-activated fibroblasts. Of note, CHIR99021 in non-activated cells failed to trigger the myofibroblast signature.

**Figure 4:**
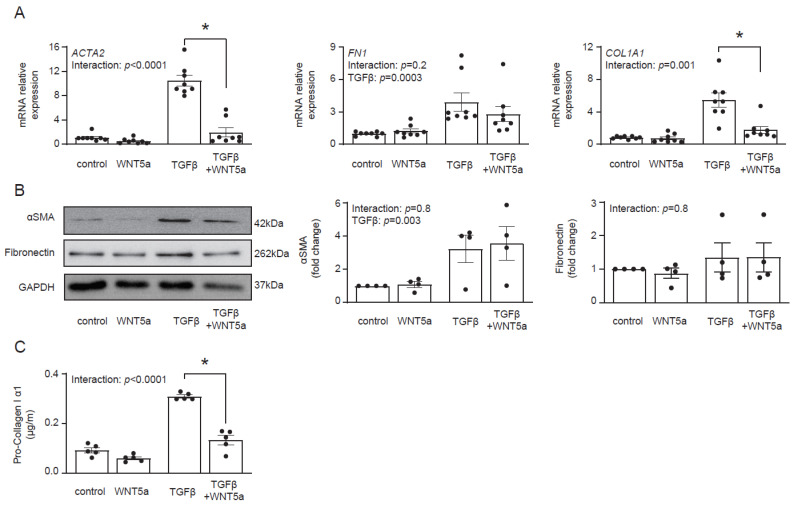
WNT5a reverses profibrotic response of TGF-β-activated cardiac fibroblasts. Panel (**A**) shows expression of profibrotic genes (*n* = 8) and panel (**B**) illustrates representative immunoblots with the respective quantifications of αSMA (*n* = 4) and fibronectin (*n* = 4) in cardiac fibroblasts stimulated with WNT5a and/or TGF-β for 72 h. Panel (**C**) shows pro-collagen I α1 levels measured in supernatants of cardiac fibroblasts treated as indicated for 5 days (*n* = 5). Bars and whiskers represent means ±SEM. Main effect of WNT5a, TGF-β and their interaction were tested using two-way ANOVA followed by Tukey’s multiple comparisons test, when the *p*-value for interaction was < 0.05, * *p* < 0.05 (post-hoc TGF-β vs. TGF-β+WNT5a).

Transcriptomics data suggested that WNT5a was not associated with profibrotic response. In fact, treatment with WNT5a suppressed TGF-β-induced expression of *ACTA2* and *COL1A1* (Figure 4A). On a protein level, however, we found no difference in αSMA levels between cells treated with TGF-β and with TGF-β and WNT5a (Figure 4B). Instead, WNT5a suppressed secretion of pro-collagen I α1 in TGF-β-activated fibroblasts (Figure 4C). Collectively, these data indicate that specific activation of the WNT/β-catenin pathway promotes fibroblast-to-myofibroblast transition induced by TGF-β.

### 2.4. WNT3a Boosts TAK1 Signaling and IL-11 Production in Activated Cardiac Fibroblasts

**Figure 5:**
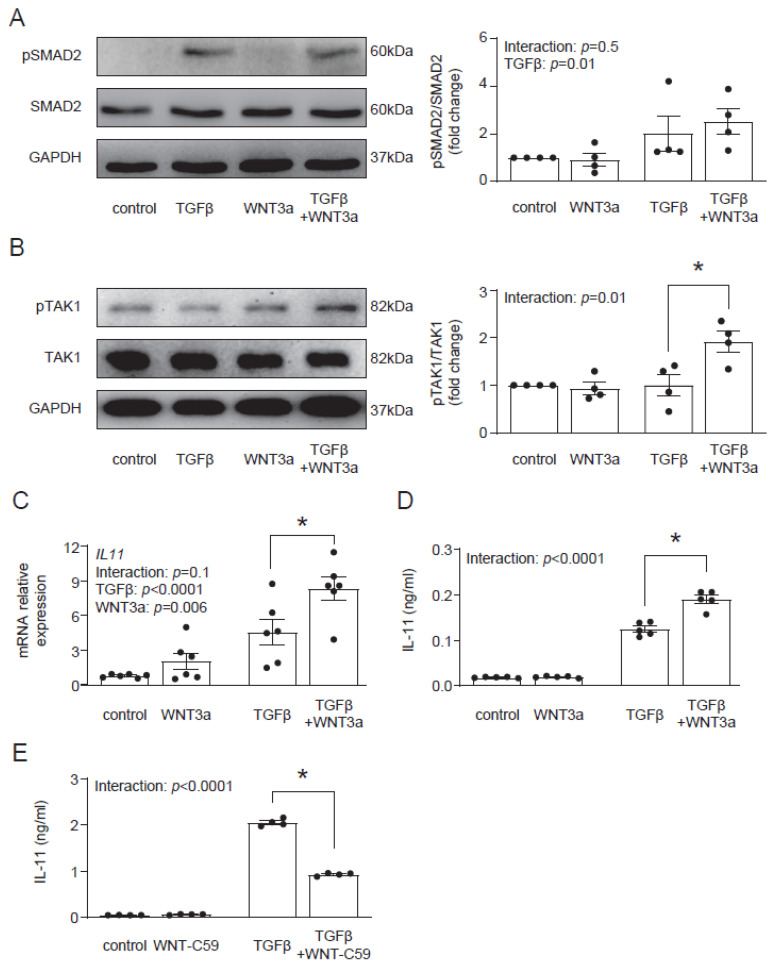
WNT3a enhances TAK1 activation and IL-11 production in TGF-β-activated fibroblasts. Representative immunoblots and the respective quantification of p-SMAD2/SMAD2 (**A**) (*n* = 4) and p-TAK1/TAK1 (**B**) (*n* = 4) in cardiac fibroblasts stimulated with Wnt3a and/or TGF-β for 1 h. Panel (**C**) indicates *IL11* gene expression (24 h *n* = 6) and panel (**D**) secreted IL-11 protein level in supernatants (72 h *n* = 5) of cardiac fibroblast stimulated as indicated. Panel (**E**) shows secreted IL-11 proteins in supernatants (*n* = 4) of cardiac fibroblast stimulated with or without TGF-β for 24 h in the presence or absence of the WNT secretion blocker WNT-C59. Bars and whiskers represent means ±SEM. Main effect of WNT3a or WNT-C59, TGF-β and their interaction were tested using two-way ANOVA followed by Tukey’s multiple comparisons test, when the *p*-value for interaction was ≤ 0.1; * *p* < 0.05 (post-hoc TGF-β vs. TGF-β+WNT3a or TGF-β vs. TGF-β+WNT-C59).

In the next step, we aimed to identify the molecular mechanism by which WNT3a enhances the profibrotic TGF-β response. In the canonical response, TGF-β activates Smad-dependent signaling. In contrast to TGF-β, WNT3a did not induce phosphorylation of Smad2 and co-stimulation with WNT3a and TGF-β showed similar activation of Smad2 to stimulation with TGF-β alone (Figure 5A). Treatment with TGF-β activates not only Smad-dependent, but also non-canonical Smad-independent pathways, such as TAK1 signaling. Even though WNT3a alone failed to activate TAK1, we observed that in the presence of TGF-β, WNT3a effectively enhanced phosphorylation of TAK1 (Figure 5B). These results suggest that WNT3a is implicated in strengthening the non-canonical arm of the TGF-β signaling in cardiac fibroblasts.

Recently, IL-11 has been implicated in profibrotic TGF-β responses in cardiac fibroblasts [17]; therefore, we addressed whether WNT3a could regulate IL-11. We found that although WNT3a alone did not regulate production and secretion of IL-11, it enhanced IL-11 production in TGF-β-activated cells (Figure 5C). As TGF-β induces WNT secretion in profibrotic responses, we studied how the blockade of WNT secretion with the porcupine inhibitor WNT-C59 affected TGF-β-induced IL-11 production. We found that treatment with WNT-C59 reduced IL-11 levels in TGF-β-activated cardiac fibroblasts (Figure 5D). These results indicate that canonical WNT signaling controls TGF-β-dependent IL-11 production.

### 2.5. IL-11 Mediates Profibrotic WNT3a-Mediated Response in Activated Cardiac Fibroblasts

**Figure 6:**
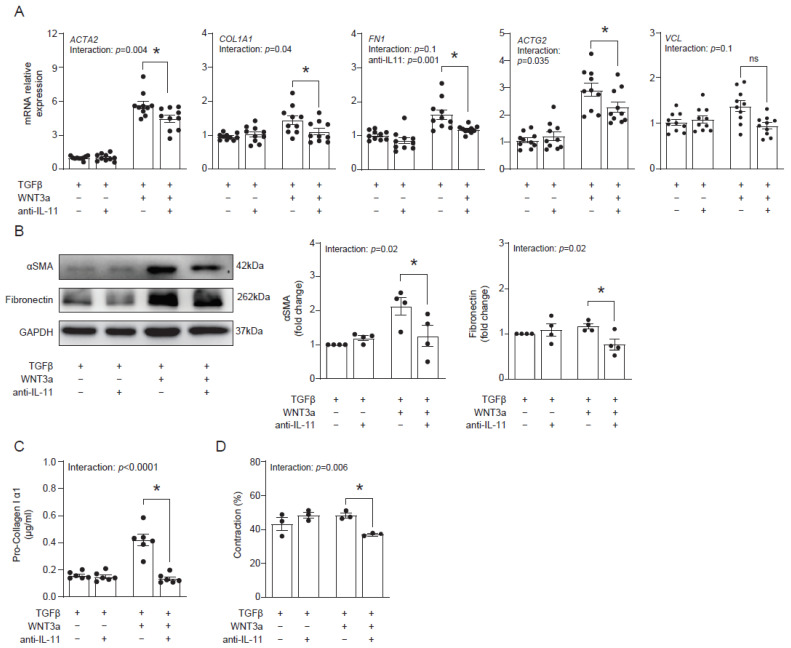
IL-11 contributes to profibrotic response enhanced by WNT3a. Human cardiac fibroblasts were cultured with TGF-β and stimulated with WNT3a in the presence or absence of an anti-IL-11 neutralizing antibody for 72 h. Panel (**A**) presents mRNA levels of profibrotic genes after indicated treatment (*n* = 10). Panel (**B**) shows quantification of αSMA and fibronectin (*n* = 4) and the representative immunoblots. Pro-collagen I α1 levels measured in supernatants (*n* = 6) are presented in panel (**C**). Panel (**D**) shows quantification of gel contraction (*n* = 3) containing cardiac fibroblasts pre-treated as indicated. Bars and whiskers represent means ±SEM. The main effect of WNT3a-TGF-β, the anti-IL11 antibody and their interaction were tested using two-way ANOVA followed by Tukey’s multiple comparisons test, when the *p*-value for interaction was ≤ 0.1; * *p* < 0.05 (post-hoc TGF-β+WNT3a vs. TGF-β+WNT3a+anti- IL11).

Our data suggested that WNT3a-dependent production of IL-11 might contribute to profibrotic responses in cardiac fibroblasts. To address this hypothesis, we blocked IL-11 activity with an anti-IL-11 neutralizing antibody in cardiac fibroblasts activated with TGF-β and WNT3a and analyzed their fibrotic profile. Indeed, the neutralizing anti-IL-11 antibody effectively suppressed transcription of profibrotic genes *ACTA2*, *COL1A1*, *FN1* and *ACTG2* in cells co-stimulated with TGF-β and WNT3a (Figure 6A). In line with regulation of *ACTA2* and *FN1* transcripts, inhibition of IL-11 reduced protein levels of αSMA and fibronectin (Figure 6B). Furthermore, inhibition of IL-11 in TGF-β-activated and WNT3a-boosted cardiac fibroblasts reduced secretion of pro-collagen I α1 (Figure 6C) and suppressed their contractile properties (Figure 6D). Collectively, obtained data confirmed the profibrotic activity of IL-11 produced by cardiac fibroblasts activated with TGF-β and WNT3a.

## 3. Discussion

Activation of cardiac fibroblasts and their transition into the myofibroblast phenotype plays a key role in cardiac fibrosis. TGF-β and WNT ligands have been identified as important profibrotic factors involved in this process, however, little is known about molecular cross-talk between these two pathways in cardiac fibrogenesis. Our data showed that specific activation of WNT/β-catenin pathways (either with WNT3a or with GSK3β inhibitor CHIR99021) promoted the fibroblast-to-myofibroblast transition, mainly by enhancing TGF-β-induced IL-11 production. In particular, WNT/β-catenin signaling specifically promoted formation of functional contractile stress fibers, an important feature of mature myofibroblasts. Previously, we showed that cardiac fibroblasts produced profibrotic WNTs upon treatment with TGF-β [29]. Here, we confirmed that also exogenous canonical WNT3a could promote fibrotic processes in cardiac fibroblasts. This is not surprising because WNT ligands are molecules that exert their signaling functions through the extracellular space. Of note, WNT secretion is a tightly regulated process resulting in controlled secretion either of soluble WNT proteins or WNTs incorporated into extracellular vesicles [29,30].

Previous studies highlighting the importance of WNT/β-catenin pathway in cardiac fibrosis were commonly based on loss-of-function approaches [16,24]. Our findings not only complement published results by gain-of-function data, but also point to the involvement of multiple profibrotic pathways in the fibroblast-to-myofibroblast transition. In fact, the inability of WNT3a to trigger fibrotic responses in non-activated fibroblasts (i.e., in the absence of TGF-β) was an important finding of this study. On a molecular level, WNT3a, unlike TGF-β, was unable to activate the Smad pathway. In contrast to our findings, treatment of mouse cardiac fibroblasts with recombinant WNT3a (in the absence of any other stimulus) activated the myofibroblast phenotype by stimulating TGF-β production and activating Smads [31]. These differences might result from differential activation status of the fibroblasts due to culture conditions, isolation procedures and/or cellular origins. Less likely, it might originate from the difference in WNT/β-catenin signaling between mouse and human cells. Nevertheless, results of both studies point to cooperative activity of TGF-β and WNT3a in the cardiac fibroblast-to-myofibroblast transition. We think that the efficient phenotypic switch to myofibroblasts requires co-activation of multiple molecular pathways including Smad, β-catenin and TAK1 in cardiac fibroblasts. This multiple pathway co-activation hypothesis is further strengthened by the data showing that blockade of one of these pathways protects cardiac fibroblasts from activation and the heart from fibrosis [12,23,32].

In our experiments, WNT3a showed no effect on activation of the Smad pathway, but enhanced TAK1 signaling in the presence but not in the absence of TGF-β. These findings may suggest that once Smad signaling is on, the output of the β-catenin pathway regulates profibrotic responses in cardiac fibroblasts. It should be noted, however, that in addition to Smads, TGF-β activates non-classical responses dependent on mitogen-activated protein kinases, such as Erk1/2, the JNKs and the p38 isoforms [14]. Regulation of these non-classical TGF-β pathways by WNT3a in cardiac fibroblasts remains, however, unknown. Regardless of the need for activation of the classical (Smad-dependent) or non-classical arm of TGF-β response, the β-catenin pathway seems to play a central role in regulating the degree of myofibroblast differentiation and thereby fibrosis. This study identifies TAK1 pathway as a molecular target of β-catenin in activated fibroblasts. Interestingly, previous data showed that inhibition of TAK1 signaling blocked the activation of the β-catenin pathway in TGF-β-activated cells [23]. In the light of both findings, a β-catenin–TAK1 feedback loop mechanism could be suggested. At this point, one needs to mention that TGF-β activates β-catenin and our recent study indicated critical involvement of endogenous angiotensin II signaling in this process. Interestingly, cells lacking the angiotensin II receptor 1 showed impaired TAK1 activation following TGF-β treatment [33]. All these data point to the β-catenin–TAK1 positive feedback loop as a novel mechanism in TGF-β-activated cells.

Our study identified canonical WNT as a new regulator of profibrotic IL-11 in TGF-β-activated cardiac fibroblasts. Previous studies showed that IL-11 is a downstream effector of TGF-β-mediated fibrosis in the heart, lungs, aorta and kidneys [17,26,28]. In line with previous findings, we observed that the blockade of IL-11 suppressed profibrotic signature in activated myofibroblasts. As in the case of profibrotic response, WNT3a in the absence of TGF-β was insufficient to regulate IL-11. We consider that IL-11 is directly regulated by β-catenin signaling at the transcriptional level, but its expression requires co-activation of other TGF-β-dependent pathways. Of note, IL-11 regulates Erk1/2 and thereby accelerates one of the non-classical arms of TGF-β signaling. Collectively, these data underline a superior role of TGF-β in cardiac fibrosis and the complexity of the downstream mechanisms. The β-catenin–TAK1 axis triggering IL-11 production seems to represent a major molecular mechanism of TGF-β-dependent fibrosis. Regulation of IL-11 by the β-catenin pathway has not been reported in fibrosis, however, cooperation of TGF-β and WNT in regulating IL-11 was observed in intestinal tumor models [34].

In opposition to profibrotic activity of the canonical WNT3a, a non-canonical WNT5a suppressed the fibroblast-to-myofibroblast transition induced by TGF-β. It should be noticed that WNT5a does not activate the β-catenin pathway and therefore is unable to promote the myofibroblast phenotype. In line with our findings, anti-fibrotic but also anti-angiogenic effects of WNT5a have been also reported in a model of TGF-β-dependent peritoneal injury [35]. Furthermore, published data demonstrated WNT5a controlling IL-6 production in human cardiac fibroblasts [29,36]. It seems that WNT5a is switching the phenotype of cardiac fibroblasts from profibrotic towards proinflammatory. Interestingly, despite IL-11 and WNT5a showing opposite effects on the activation of cardiac fibroblast, the Erk1/2 pathway was identified as a key downstream signal for both of them [17,37]. It suggests that Erk1/2 might act in a pro- or anti-fibrotic way depending on the presence of specific co-activators or the status of activated cells, but any other alternative mechanism cannot be excluded.

Cardiac fibrosis often develops as a response to pathogenic conditions in hearts, such as hypoxia, mechanical or oxidative stress and inflammation. In this light, cell culture models are helpful to study specific molecular mechanisms but do not reflect complexity of fibrogenesis, which in the human heart is not only multifactorial, but is also a long-lasting process. On molecular level, TGF-β signaling is considered as a master profibrotic trigger in the heart, however, there are other secreted biomolecules, such as angiotensin II, damage associated molecular pattern molecules, endothelin-1, connective tissue growth factor and platelet-derived growth factor, cytokines IL-1 or IL-17, that can induce or exacerbate cardiac fibrosis. It should be acknowledged that our data were obtained with fetal cardiac fibroblasts showing premature differentiation status. However, cardiac fibroblasts isolated from adult human hearts once cultured in vitro usually become activated, show the myofibroblast phenotype and respond poorly to TGF-β, and therefore they are not a useful model to study the TGF-β-dependent fibroblast-to-myofibroblast transition. Furthermore, the relevance of in vitro-identified molecular mechanisms needs to be verified in animal models and in humans.

A growing body of evidence indicates elevated levels of multiple WNT ligands in the effected heart [16,32]. Thus, a balance between canonical and non-canonical WNT signaling might be decisive for the fate of activated cardiac fibroblasts in the injured heart and eventually for development of cardiac fibrosis. Accumulating data strongly indicate that the canonical β-catenin pathway is critically involved in pathogenesis of cardiac fibrosis. Herein, by using a model of human cardiac fibroblasts, we elucidated an interplay between TGF-β, WNT/β-catenin and IL-11 and proposed a novel mechanism controlling cardiac fibrosis. This study adds molecular knowledge to our understanding of profibrotic mechanisms in the heart, which is essential for the development of effective cardioprotective therapies targeting cardiac fibrosis.

## 4. Materials and Methods

### 4.1. Cell Cultures and Stimulations

Primary fetal human cardiac cells were originally obtained from Cell Applications (Cell Applications, San Diego, CA, USA). L-WNT3a (overexpressing WNT3a), L-WNT5a (overexpressing WNT5a) and L (control line for L-WNT3a and L-WNT5a) cell lines were obtained from ATCC (ATCC, Manassas, VA, USA). The AP-1 reporter cardiac fibroblasts were described previously [29]. Cells were cultured in the Dulbecco’s modification of Eagle medium (DMEM, Corning, New York, NY, USA) and supplemented with 10% FBS (EURx, Gdansk, Poland), 1:100 penicillin/streptomycin, 100 mM nonessential amino acids (all Corning, New York, NY, USA) and 50 mM β-mercaptoethanol (Sigma-Aldrich, Taufkirchen, Germany). L-WNT3a and L-WNT5a cell lines were selected with medium containing G418 (Sigma-Aldrich). Human cardiac fibroblasts were cultured in up to 12 passages. Cells were passaged using standard protocol with 0.25% trypsin (Corning) and seeded 24 h before experiments. All cells used in this study were mycoplasma-free. Cell culture supernatants from L-, L-WNT3a and L-WNT5a cultured for 48 h were collected, filtered (pore size 0.2 µm, Carl Roth, Karlsruhe, Germany) and used fresh for cardiac fibroblasts stimulation. Cells were stimulated with 10 ng/mL of TGF-β (Peprotech, Cranbury, NJ, USA) or with 1 µM CHIR99021 (CHIR, Tocris, Bristol, UK) in the presence or absence of 10 ng/mL TGF-β for 72 h. To block WNT secretion, cells were treated with 0.1 µM Wnt-C59 (Tocris) with or without 10 ng/mL TGF-β for 24 h. Neutralization of IL-11 was performed with 0.5 µg/mL of an anti-IL-11 antibody (clone: 226226, R&D systems, Minneapolis, MN, USA) for 72 h.

### 4.2. Immunofluorescence

After stimulation, cells were fixed with 4% paraformaldehyde for 5 min at room temperature, permeabilized with 0.01% Triton X-100 for 5 min and blocked with 5% BSA in PBS for 20 min. Specimens were incubated with primary mouse anti-β-catenin (1:500, clone CAT-5H10, Thermo Fisher Scientific, Waltham, MA, USA) and an anti-αSMA antibody (1:200, clone 1A4, Biolegend, San Diego, CA, USA) at room temperature, followed by the secondary AlexaFluor555 goat anti-mouse antibody (1:8000) and phalloidin (1:1000, all Thermo Fisher Scientific) at room temperature for 1 h. Hoechst (1:1000, Thermo Fisher Scientific) was used to label the nuclei. Immunofluorescence was analyzed using Leica DM5500 B fluorescence microscope with DFC365 FX camera (Leica Microsystems, Wetzlar, Germany).

### 4.3. Quantitative RT-PCR

Total RNA was extracted with Trizol Reagent (Thermo Fisher Scientific). 150 ng or 250 ng of total RNA was used to synthesize complementary DNA using NG dART RT Kit (EURx, Gdansk, Poland). Quantitative real-time PCR was performed using SYBR Green PCR Master Mix (EURx) and oligonucleotides complementary to transcripts of the analyzed genes using the Quant Studio 7 Real-Time PCR system (Applied Biosystems, Foster City, CA, USA). The following oligonucleotides were used in this study: AXIN2: 5′-TAACCCCTCAGAGCGATGGA-3′ and 5′-CCTCCTCTCTTTTACAGCAGGG-3′, TCF7: 5′-CCAAGAATCCACCACAGGAGG-3′ and 5′-GCCTAGAGCACTGTCATCGG-3′, JUN: 5′-GAGCTGGAGCGCCTGATAAT-3′ and 5′—CCCTCCTGCTCATCTGTCAC-3′, ATF2: 5′-GTGAATTCTGCCAGGCAATAC-3′ and 5′-CCTCGTTGGTAAAACGCTGG-3′, ACTA2: 5′-GACAATGGCTCTGGGCTCTGTAA-3′ and 5′-ATGCCATGTTCTATCGGGTACTT-3′, IL11: 5′ TCG AGT TTC CCC AGA CCC TC-3′ and 5′-GAA TCC AGG TTG TGG TCC CC-3′, ACTG2: 5′-GCAAGCGAGGGATCCTAACTC-3′ and 5′- AGGAGTGGTGCCAGATCTTCTC-3′ COL1A1: 5′-CAGCCGCTTCACCTACAGC-3′ and 5′-TTTTGTATTCAATCACTGTCTTGC-3′, FN1: 5′-TGCCACTGTTCTCCTACGTGG-3′ and 5′- GGAGAATTCAAGTGTGACCCTCATG-3′, VCL: 5′- AATGGTCCAGCAAGCCGGG-3′ and 5′- TCGTCTTCCATGTCAGGGGG-3′, GAPDH: 5′-GGGAAGCTTGTCATCAATGGA-3′ and 5′-TCTCGCTCCTGGAAGATGGT-3′. Transcript levels of GAPDH were used as an endogenous reference and relative gene expression was calculated using the 2−∆∆Ct method.

### 4.4. Luciferase Assay

Luciferase expression in the AP-1 reporter fibroblasts was assessed using the Bright-Glo Luciferase Assay System (Promega, Madison, WI, USA) according to the manufacturer’s protocol. Bioluminescence was measured by a SyneryHT plate reader (BioTek, Winooski, VT, USA) or Wallac Victor2 1420 Multilabel counter (Perkin-Elmer, Waltham, MA, USA).

### 4.5. RNA-Seq Analysis

Total RNA from three biological independent experiments of cardiac fibroblasts were sent to Genewiz for preparation in RNA-Seq libraries. Libraries were run with Illumina, strand-specific RNA-Seq with PolyA selection and Illumina NovaSeq 2 × 150 bp sequencing, 10M read pairs. Raw reads were mapped to the Ensembl human GRch38 reference genome using the STAR aligner v.2.5.2b. Unique gene hit counts were calculated by using feature Counts from the Subread package v.1.5.2. After extraction of gene hit counts, the gene hit counts table was used for downstream differential expression analysis. Comparison of the gene expression between the groups was performed using DESeq2. The Wald test was used to generate *p* values and log2 fold changes. Genes with an adjusted *p* value < 0.05 and absolute log2 fold change > 1 were called as differentially expressed genes for each comparison. Differentially expressed probes from respective groups are highlighted in the heatmap plot. The heatmap was generated using online tool: https://software.broadinstitute.org/morpheus/. The raw values were transformed, scaled with red representing upregulated genes and blue representing downregulated genes. The GO analyses were performed using online tool: https://tools.dice-database.org/GOnet/ for human species. The raw data file is uploaded to Gene Expression Omnibus (GEO) database with number: GSE181575.

### 4.6. Western Blotting

For protein expression analysis, a conventional SDS–PAGE followed by protein blotting strategy was performed as described previously [29]. The following antibodies were used: rabbit anti-GAPDH (1:5000, clone GA1R, Thermo Fisher Scientific), rabbit anti-αSMA (1:800, clone 1A4, Biolegend), mouse anti-fibronectin (1:700, polyclonal, Abcam), rabbit anti -TAK (polyclonal 1:1000), rabbit anti-phospho-TAK (polyclonal, 1:1000), anti-Smad2 (clone D43B4, 1:1000) and anti-phosho-Smad2 (clone 138D4, 1:1000, all Cell Signaling Technology, Danvers, MA, USA). Protein abundance was normalized to GAPDH levels. Results were analyzed with ImageJ software (NIH, Bethesda, MD, USA) according to methods for Western Blot densitometry band quantification through image analysis software with background subtraction [38].

### 4.7. ELISA

Type I collagen was measured in cell culture supernatants using Human Procollagen I alpha 1 DuoSet ELISA (R&D Systems) and IL-11 was measured in cell culture supernatants using IL-11 Human ELISA kit (Thermo Fisher Scientific) according to the manufacturer’s protocol.

### 4.8. Collagen Contraction Assay

After stimulations, cells were trypsinized, counted and mixed with the Collagen Gel Working Solution prepared according to manufacturer’s protocol (Cell Biolabs Inc., San Diego, CA, USA). The solution was added to 24 well plates and allowed to polymerize for 1 h at 37 °C. Fresh growth media was added to the solidified collagen gels and plates were returned to the incubator. After 24 h, the surface of contraction was released and contraction was monitored over the next 24 h. Next the surface area of contracted gels was imaged using a ChemiDoc instrument (Bio-Rad) and measured using ImageJ software (NIH).

### 4.9. Statistical Analysis

Experimental results are presented as mean± SEM. Raw data normally distributed and data with homogenous variance (Brown–Forsythe test) were compared using a one-way ANOVA followed by Dunn’s post-hoc test or two-way ANOVA followed by Tukey’s post-hoc test for the indicated comparisons. All analyses were computed using GraphPad Prism 9.1.1 software. Differences were considered as statistically significant for *p* < 0.05.

## Data Availability

All relevant data are within the paper and its Supplementary Materials files. The data that support the findings of this study are available in Gene Expression Omnibus (GEO) database with number: GSE181575.

## Supplementary Materials

The following are available online at https://www.mdpi.com/article/10.3390/ijms221810072/s1. Figure S1: Heat map of all differentially expressed genes (fold change > 2.0 and *p* < 0.05) in fibroblasts treated with TGF-β versus control. Figure S2: Heat map of all differentially expressed genes (fold change > 2.0 and *p* < 0.05) in fibroblasts treated with WNT3a/TGF-β versus TGF-β alone.
