# Supplementary material for: WNT/β-Catenin Signaling Promotes TGF-β-Mediated Activation of Human Cardiac Fibroblasts by Enhancing IL-11 Production
Source: Int J Mol Sci. 2021 Sep 17;22(18):10072. doi: 10.3390/ijms221810072 (PMC8468519; doi:10.3390/ijms221810072)

Supp Fig. S1

Heat map of all differentially expressed genes (fold change>2.0 and p<0.05) in fibroblasts treated with TGF-β versus control.

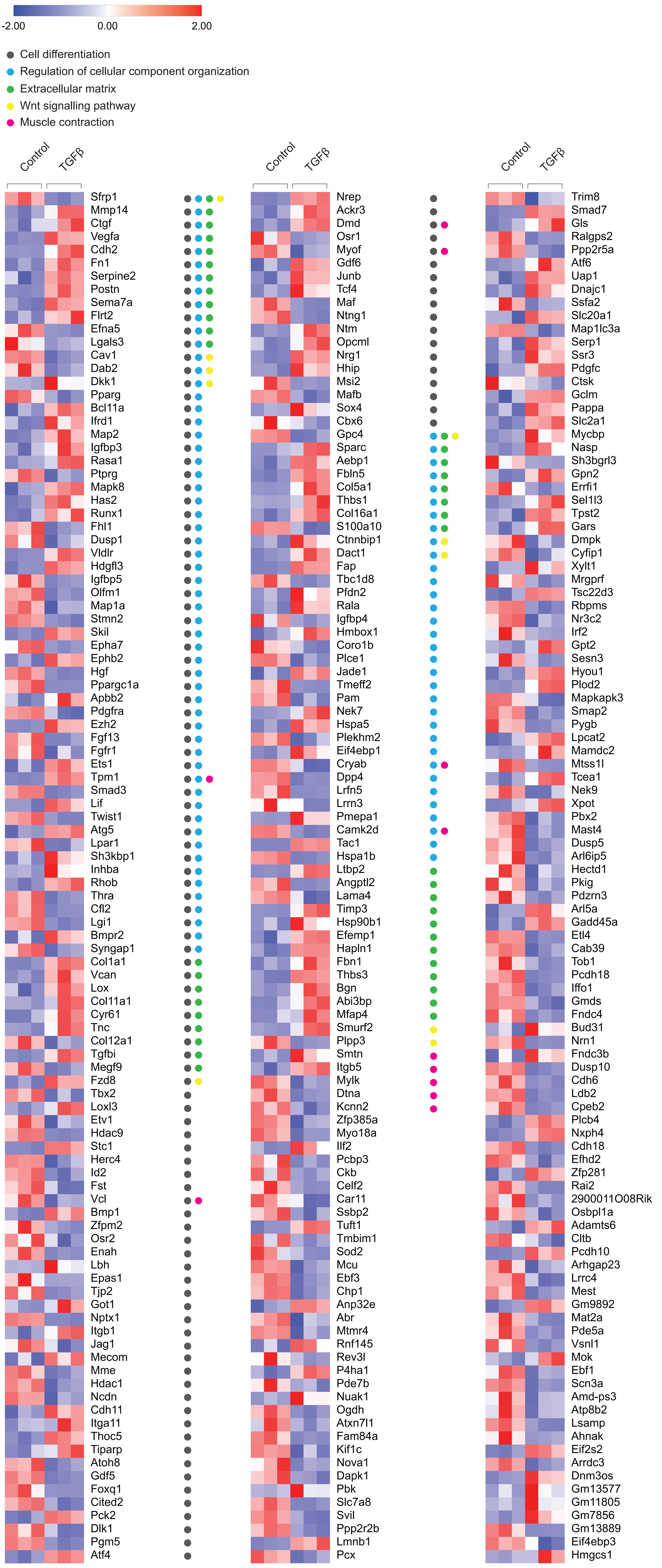

Supp Fig. S2

Heat map of all differentially expressed genes (fold change>2.0 and p<0.05) in fibroblasts treated with WNT3a/TGF-β versus TGF-β alone.

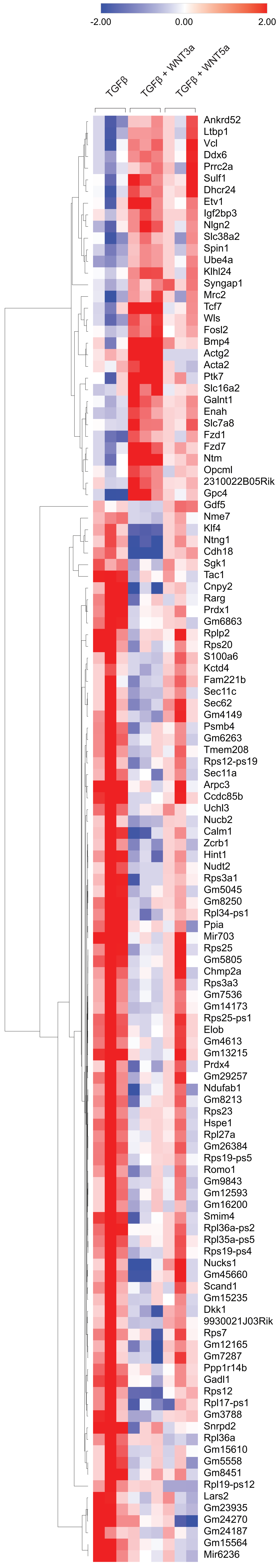

Supplement: Supplementary file 1 [file ijms-22-10072-s001.zip › ijms-1338230-supplementary.pdf]
